# Evaluation of a pan-*Leishmania* SL RNA qPCR assay for parasite detection in laboratory-reared and field-collected sand flies and reservoir hosts

**DOI:** 10.1186/s13071-020-04141-y

**Published:** 2020-06-01

**Authors:** Myrthe Pareyn, Rik Hendrickx, Nigatu Girma, Sarah Hendrickx, Lieselotte Van Bockstal, Natalie Van Houtte, Simon Shibru, Louis Maes, Herwig Leirs, Guy Caljon

**Affiliations:** 1 Evolutionary Ecology Group, University of Antwerp, Wilrijk, Belgium; 2 Laboratory of Microbiology, Parasitology and Hygiene, University of Antwerp, Wilrijk, Belgium; 3 Biology Department, Arba Minch University, Arba Minch, Ethiopia

**Keywords:** Spliced leader RNA, Kinetoplast DNA, Nucleic acid extraction, Sand fly, Reservoir, Real-time PCR

## Abstract

**Background:**

In eco-epidemiological studies, *Leishmania* detection in vectors and reservoirs is frequently accomplished by high-throughput and sensitive molecular methods that target minicircle kinetoplast DNA (kDNA). A pan-*Leishmania* SYBR green quantitative PCR (qPCR) assay which detects the conserved spliced-leader RNA (SL RNA) sequence was developed recently. This study assessed the SL RNA assay performance combined with a crude extraction method for the detection of *Leishmania* in field-collected and laboratory-reared sand flies and in tissue samples from hyraxes as reservoir hosts.

**Methods:**

Field-collected and laboratory-infected sand fly and hyrax extracts were subjected to three different qPCR approaches to assess the suitability of the SL RNA target for *Leishmania* detection. Nucleic acids of experimentally infected sand flies were isolated with a crude extraction buffer with ethanol precipitation and a commercial kit and tested for downstream DNA and RNA detection. Promastigotes were isolated from culture and sand fly midguts to assess whether there was difference in SL RNA and kDNA copy numbers. Naive sand flies were spiked with a serial dilution of promastigotes to make a standard curve.

**Results:**

The qPCR targeting SL RNA performed well on infected sand fly samples, despite preservation and extraction under presumed unfavorable conditions for downstream RNA detection. Nucleic acid extraction by a crude extraction buffer combined with a precipitation step was highly compatible with downstream SL RNA and kDNA detection. Copy numbers of kDNA were found to be identical in culture-derived parasites and promastigotes isolated from sand fly midguts. SL RNA levels were slightly lower in sand fly promastigotes (ΔCq 1.7). The theoretical limit of detection and quantification of the SL RNA qPCR respectively reached down to 10^−3^ and 10 parasite equivalents. SL RNA detection in stored hyrax samples was less efficient with some false-negative assay results, most likely due to the long-term tissue storage in absence of RNA stabilizing reagents.

**Conclusions:**

This study shows that a crude extraction method in combination with the SL RNA qPCR assay is suitable for the detection and quantification of *Leishmania* in sand flies. The assay is inexpensive, sensitive and pan-*Leishmania* specific, and accordingly an excellent assay for high-throughput screening in entomological research.
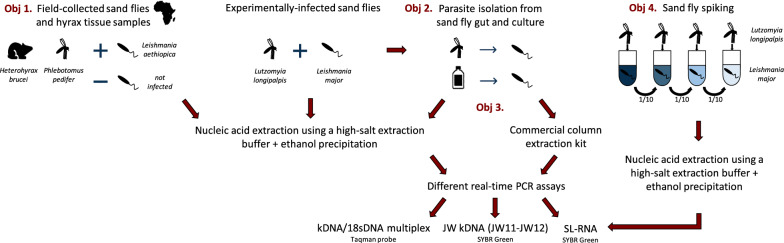

## Background

Leishmaniasis is a vector-borne disease caused by protozoans of the genus *Leishmania*, which are transmitted during the blood-feeding of female phlebotomine sand flies. The infection can be manifested in three major clinical forms, cutaneous (CL), mucocutaneous, and visceral leishmaniasis [1].

In Ethiopia, *Leishmania aethiopica* is the predominant species causing CL and its vectors are *Phlebotomus longipes* and *P. pedifer* [2–4]. Hyraxes (*Heterohyrax brucei* and *Procavia capensis*) have been found asymptomatically infected with *L. aethiopica* in large numbers, indicating that they are major animal reservoirs in Ethiopia [3–5].

For eco-epidemiological research, which is often covering large sample sizes, there is a need for low-cost, sensitive, high-throughput methods to identify and quantify *Leishmania* parasites in (potential) vectors and hosts [6]. The golden standard for parasite detection in sand flies and animal tissues is microscopy examination. This method allows to confirm the presence of viable parasites, but is time consuming and requires a substantial level of expertise [7]. These drawbacks resulted in a shift towards sample screening with molecular assays. Procedures generally start with nucleic acid extraction for which efficient, but expensive kits are commercially available. Low-cost methods, like organic (i.e. phenol-chloroform) or chelex extractions, are widely utilized, but have disadvantages. The former method is very time consuming and often involves toxic chemicals while the latter only yields low amounts of genomic DNA [8]. Extraction approaches with lysis buffers containing SDS, EDTA, Tris-HCl and NaCl have been applied successfully to various tissues [9], although this crude procedure may lead to inhibition in downstream molecular applications [8].

A variety of (real-time) PCR methods targeting different gene fragments has been described, many of which remain to be validated on multiple *Leishmania* species and different tissues, or have issues regarding quantification [10, 11]. The most commonly used PCR assay for *Leishmania* detection in sandflies [12, 13] and small mammals [14–16] is targeting the minicircle kinetoplast DNA (kDNA). Because of the high kDNA copy number (10^4^ minicircles per parasite), very low numbers of parasites can be detected [7]. However, the nucleotide sequence and copy number sometimes differ among *Leishmania* species, impeding consistent quantification [17, 18]. Another concern is that it sometimes results in false positive assay results due to its high sensitivity, even though all preventive measures to avoid contamination are taken [19–21].

Few studies investigated the use of RNA targets for parasite detection, although these may be more informative than DNA targets given the ability to discriminate viable parasites [22]. Recently, a pan-*Leishmania* SYBR Green quantitative PCR (qPCR) assay has been developed, targeting the highly conserved mini-exon encoded 39 bp spliced-leader RNA (SL RNA) sequence, which shows excellent sensitivity and specificity. The assay was able to detect eight Old- and New-World *Leishmania* species with equal threshold cycle (Cq) values and was validated on tissue samples of *L. infantum-*infected hamsters, promastigote spiked human blood and blood nucleic acid extracts from visceral leishmaniasis patients. It appeared that the limit of detection (LoD) of the SL RNA qPCR was one log lower than the LoD of a TaqMan duplex assay targeting kDNA [23].

In this study, we aimed to evaluate the SL RNA qPCR assay in combination with a crude extraction procedure for detection and quantification of *Leishmania* parasites in field- and laboratory-collected (infected) sand flies and hyrax tissue samples collected in Ethiopia.

## Methods

### Parasites

The *L. major* strain MHOM/SA/85/JISH118 used in this study was cultivated *in vitro* at 26 °C in HOMEM promastigote medium (Gibco, Life Technologies, Ghent, Belgium), supplemented with 10% inactivated fetal calf serum (Invitrogen, Merelbeke, Belgium) and was sub-cultured twice weekly.

### Sand flies

*Lutzomyia longipalpis* sand flies were maintained at the insectary of the Laboratory of Microbiology, Parasitology and Hygiene, Antwerp, Belgium. The colony was kept at 25–26 °C, 75% relative humidity and 12:12 h light:dark photoperiod. A 30% sugar source was permanently provided to adult sand flies. Depending on the experiment, naive or experimentally infected *L. longipalpis* were used. For laboratory infection, the sand flies were starved 12 h prior to feeding through a chick-skin membrane on heparinized (100 U/ml blood) heat-inactivated mouse blood spiked with *L. major* procyclic promastigotes (5 × 10^6^ promastigotes/ml blood). Engorged females were separated 24 h post-blood meal and were continuously provided with 30% sugar solution.

*Phlebotomus pedifer* sand flies were captured in a previous study in Ochollo (6°11′ N, 37°41′ E), a village in southwestern Ethiopia where CL is endemic [24, 25]. Sand flies were captured between March 2017 and February 2018 using CDC light traps and sticky traps. Specimens were stored in 97% ethanol at − 20 °C until nucleic acid isolation was carried out in March 2018 (as described in ‘Sand fly nucleic acid isolation and purification’ below and in [24]). *Leishmania* DNA positive sand flies were all *P. pedifer* infected with *L. aethiopica* [24]. Nucleic acid extracts were stored at − 20 °C until analysis for the current study.

### Hyraxes

Hyraxes had been captured in Ochollo using traditional trapping methods in 2017. Nose and ear samples were collected and stored in 97% ethanol at − 20 °C until further handling. Molecular analyses revealed that all hyraxes were *H. brucei* infected with *L. aethiopica*. The original tissue samples in 97% ethanol were stored at − 20 °C until analysis [24].

### SL RNA qPCR evaluation on field and laboratory-infected sand flies and hyraxes

#### Sand fly nucleic acid isolation and purification

Experimentally infected (*L. major*) *L. longipalpis* were collected six days after infection for dissection of the thorax and abdomen (*n* = 96). Nucleic acids of these specimens were isolated with a crude extraction buffer and purified using an ethanol precipitation approach as described previously [24]. In short, individual sand fly specimens were incubated overnight in 50 µl extraction buffer (10 mM Tris-HCl pH 8, 10 mM EDTA, 0.1 % SDS, 150 mM NaCl) and 0.5 µl proteinase K (200 µg/ml) without maceration. The next day, 25 µl nuclease-free water was added and the samples were heated for 5 min at 95 °C. For nucleic acid precipitation, 20 µl of the extract was supplemented with 1/10th volume 3 M NaOAc (pH 5.6) and 2 volumes 97% ethanol (chilled at − 20 °C). This suspension was left overnight, after which the samples were centrifuged for 15 min at 21,000×*g* at 4 °C. The supernatant was removed and 500 µl chilled 70% ethanol was added, followed by centrifugation under the same conditions. The supernatant was removed, and the pellet was air-dried for 15 min in a heating block at 50 °C followed by resuspension in 20 µl nuclease-free water.

Additionally, 37 *P. pedifer* nucleic acid extracts were selected from our previous study (see section ‘Sand flies’), of which 17 were identified as *L. aethiopica* positive (kDNA and ITS1) and 20 as negative (kDNA) [24].

#### DNA/RNA extraction from hyrax samples

Seven *L. aethiopica* DNA positive (kDNA and ITS-1) and 15 negative hyrax tissue samples were selected from our previous study (see section ‘Hyraxes’) [24]. DNA and RNA were simultaneously extracted from the original tissue samples with the NucleoSpin RNA kit (Macherey Nagel, Düren, Germany) and additional reagents from the NucleoSpin RNA/DNA buffer set (Macherey Nagel) according to the manufacturer’s instructions.

#### Molecular screening

Nucleic acid extracts of the sand fly and hyrax samples were subjected to three different real-time PCR approaches, targeting four markers: (i) kDNA and *18S* DNA in a multiplex TaqMan probe assay (further referred to as ‘MP kDNA’ and ‘*18S* DNA’, respectively); (ii) kDNA in a SYBR Green assay with an alternate set of primers (‘JW kDNA’); and (iii) SL RNA in a SYBR Green assay (‘SL RNA’). The primers for the JW kDNA qPCR were adopted from Nicolas et al. [14] and the assay was carried out as explained in our previous study [24], while the other assays were performed as described by Eberhardt et al. [23]. All extracts were diluted 1:10 prior to qPCR to prevent inhibition of the polymerase enzyme. All assays were run on a Step One Plus real-time qPCR system (Applied Biosystems, Life Technologies) and the threshold was set at 1 for each qPCR.

### Copy number and comparison of extraction methods

#### Promastigote isolation from sand fly midgut and culture

We assessed whether there is a potential copy number difference of kDNA and SL RNA between parasites isolated from sand fly midguts and *in vitro* cultures in HOMEM. First, promastigotes were harvested from midguts of *L. major* experimentally infected *L. longipalpis* (see section ‘Sand flies’). Sand flies were collected six days after feeding on a blood meal containing parasites, and the midguts were dissected under a dissection microscope. Pools of midguts were macerated with a pestle in 100 µl Dulbecco’s phosphate-buffered saline (DPBS; Gibco, Thermo Fisher Scientific, Ghent, Belgium) to release the parasites. Secondly, *L. major* promastigotes from a culture were counted using a KOVA chamber to determine parasite concentration. An excess volume was taken for further washing steps. Both suspensions were washed twice in 100 µl DPBS with intermediate centrifugation steps of 1 min at 21,300×*g*. The pellet was resuspended in 100 µl DPBS. Parasite concentrations were determined in a KOVA chamber and used to prepare two replicates of 10^6^, 10^5^ and 10^4^ parasites in 20 µl DPBS from promastigotes isolated from the sand fly midguts and from culture.

#### DNA/RNA isolation and molecular screening

To determine whether the crude extraction buffer in combination with ethanol precipitation is suitable for efficient nucleic acid isolation and subsequent downstream RNA and DNA detection, nucleic acids from three different concentrations of promastigotes, isolated from either sand fly midguts or culture medium, were extracted using (i) a Nucleospin RNA kit and additional RNA/DNA buffer set (Macherey Nagel) and (ii) the crude extraction buffer and ethanol precipitation approach. For the latter, the complete volume of the nucleic acid extract was used for ethanol precipitation. The final elution volumes were equalized to ensure the same relative DNA and RNA yields for both methods. All extracts were subjected in duplicate to the qPCRs targeting JW kDNA and SL RNA.

### Contribution of RNA *versus* DNA in the SL RNA qPCR assay

Ten *L. major-*infected *L. longipalpis* nucleic acid isolates that were used for the qPCR assay comparison experiment were selected. These samples were subjected in duplicate to the SL RNA qPCR assay with and without the use of a reverse transcriptase enzyme to demonstrate how much of the fluorescent signal originates from RNA *versus* DNA in the crude sand fly extracts. For the assay without reverse transcriptase, the volume of the enzyme was replaced by nuclease-free water. The percentage of DNA detected by the SL RNA qPCR assay was calculated by $\frac{{ 1 0 0 {\text{\% }}}}{{ 2^{{ ( {\text{Cq with RT - Cq without RT)}}}} }}$ based on the assumption that each PCR cycle doubles the number of amplicons.

### Sand fly spiking

Promastigotes (*L. major*) were harvested from a stationary-phase culture (see section ‘Parasites’) and washed with DPBS. The number of promastigotes was determined in a KOVA counting chamber and the pellet was stored at − 20 °C until extraction. Naive laboratory-reared *L. longipalpis* sand flies were spiked with a 10-fold serial dilution of *L. major* promastigotes, ranging from 1.6 × 10^7^ to 1.6 × 10^−6^ parasites. The samples were extracted with the crude extraction buffer and ethanol precipitation approach, and subsequently subjected in duplicate to the SL RNA qPCR.

### Data analysis

Analyses were carried out using GraphPad Prism version 8 (GraphPad Software, La Jolla California, USA). The correlation between the Cq values of the qPCRs targeting SL RNA and the other three markers was determined by a Pearson’s correlation test. This analysis was performed using the infected field-collected sand flies because of the broad range of Cq values. A standard curve with linear regression and PCR efficiency was generated to determine the theoretical LoD and limit of quantification (LoQ) of the SL RNA qPCR.

## Results

### Evaluation of the SL RNA qPCR for *Leishmania* detection

**Fig. 1:**
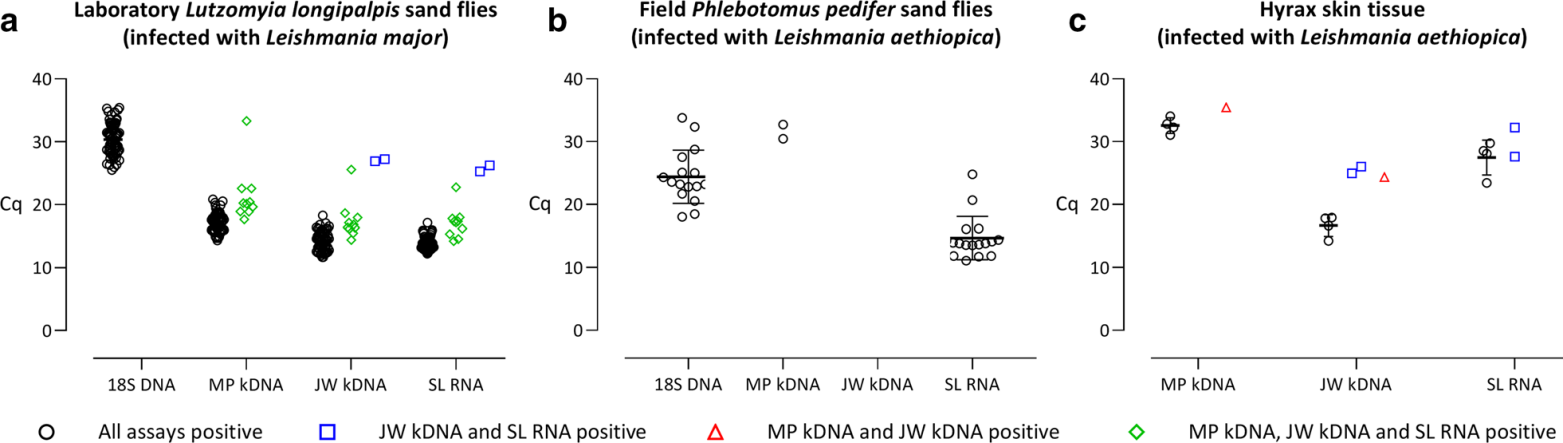
qPCR Cq values of laboratory—(**a**) and field-infected (**b**) sand flies, and infected hyrax tissue samples (**c**). Mean Cq values and error bars (standard deviation) are presented for the samples that were positive in all assays that they were tested for. Due to technical issues, the analysis of the *18S* DNA qPCR on hyrax tissue samples was not included. *Abbreviation*: Cq, quantification cycle

Of the 96 *L. major-*infected laboratory *L. longipalpis* sand flies, two samples were negative and 82 were positive assays (Fig. 1a, Additional file 1: Table S1, Additional file 2: Data S1). Among the samples that were positive by all qPCRs, the *18S* DNA marker provided the highest mean Cq value (30.3 ± 2.3), followed by MP kDNA (17.3 ± 1.4), JW kDNA (14.6 ± 1.4) and SL RNA (13.8 ± 0.9). Ten samples were not positive for the *18S* DNA marker, but were detected by all other markers. These samples had higher mean Cq values of 21.4 ± 4.4, 17.5 ± 3.1) and 17.1 ± 2.4 for the MP kDNA, JW kDNA and SL RNA markers, respectively. Two sand fly specimens with the highest Cq values for the JW kDNA (27.1 ± 0.2) and SL RNA (25.7 ± 0.7) qPCRs were not positive for the MP kDNA target. Overall, the JW kDNA and SL RNA qPCRs provided concordant results on the laboratory-infected sand flies.

Among the field-collected, ethanol stored sand fly specimens, 20 were negative and 17 positive for all assays (Fig. 1b, Additional file 1: Table S1, Additional file 3: Data S2). Mean Cq values of the JW kDNA and SL RNA qPCRs were similar (13.7 ± 3.9 and 14.7 ± 3.4, respectively) and consistently lower than the Cq values obtained by the duplex assay (*18S* DNA: 24.2 ± 4.2 and MP kDNA: 22.9 ± 4.2). The difference in Cq values between the MP kDNA and JW kDNA markers was larger for *P. pedifer* infected with *L. aethiopica* than for *L. longipalpis* infected with *L. major* (Fig. 1a *versus* b).

Seven out of 22 long-term stored hyrax tissue samples tested positive for two or more markers (Fig. 1c, Additional file 1: Table S1, Additional file 4: Data S3). Four samples were positive in all assays, resulting in the lowest Cq values for JW kDNA (16.6 ± 1.7), compared to SL RNA (27.5 ± 2.8) and MP kDNA (32.6 ± 1.2). Two samples with high Cq values in the JW kDNA and SL RNA qPCRs were negative for the MP kDNA marker, while one sample was positive for the MP kDNA and JW kDNA targets with high Cq values, but not by the SL RNA qPCR.

**Fig. 2:**
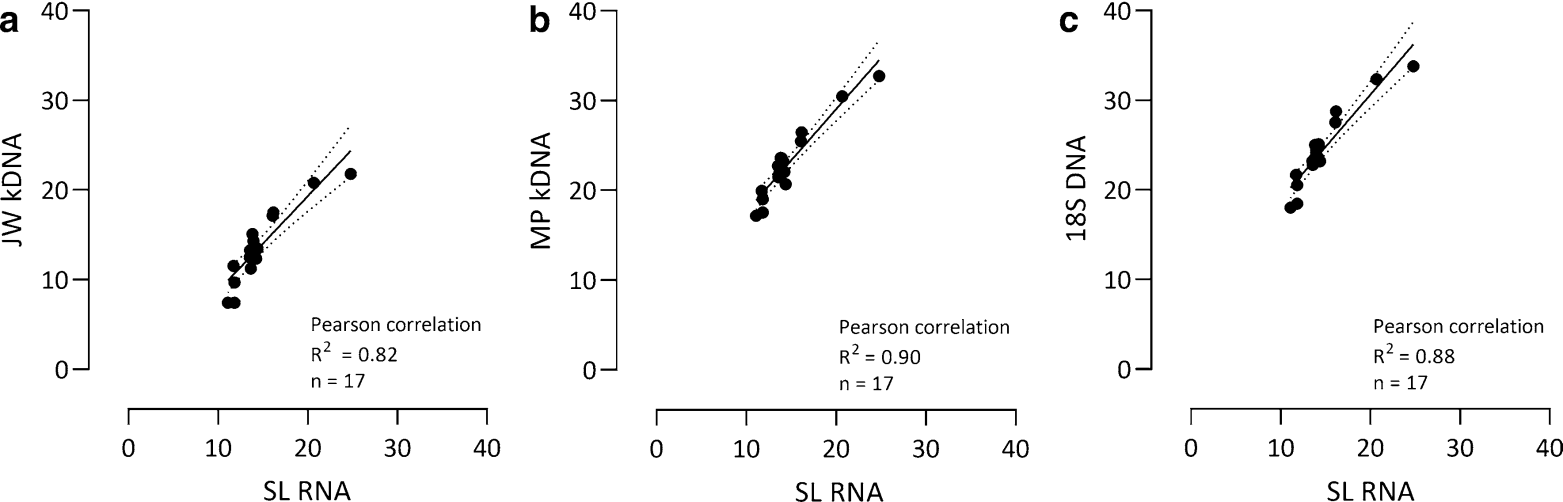
Correlation between Cq values obtained by the different qPCR assays. Correlations between Cq values of the SL RNA qPCR and the JW kDNA qPCR (**a**), MP kDNA qPCR (**b**) and *18S* DNA qPCR (**c**). Pearson’s correlation analysis of the results obtained with the different assays. Linear regression and 95% confidence intervals (dotted lines) are shown in the graphs. *Abbreviation*: Cq, quantification cycle

Overall, Pearson’s correlation showed that the Cq values for the SL RNA target correlated quite well with the Cq values of the JW kDNA (Fig. 2a; *R*^2^ = 0.82, *n* = 17), MP kDNA (Fig. 2b; *R*^2^ = 0.90, *n* = 17) and *18S* DNA markers (Fig. 2c; *R*^2^ = 0.88, *n* = 17) based on field-collected sand flies. For all comparisons, the confidence intervals increased towards the higher Cq values, which could be due to slight inhibition of the SL RNA qPCR.

### Extraction method comparison and copy number difference

**Fig. 3:**
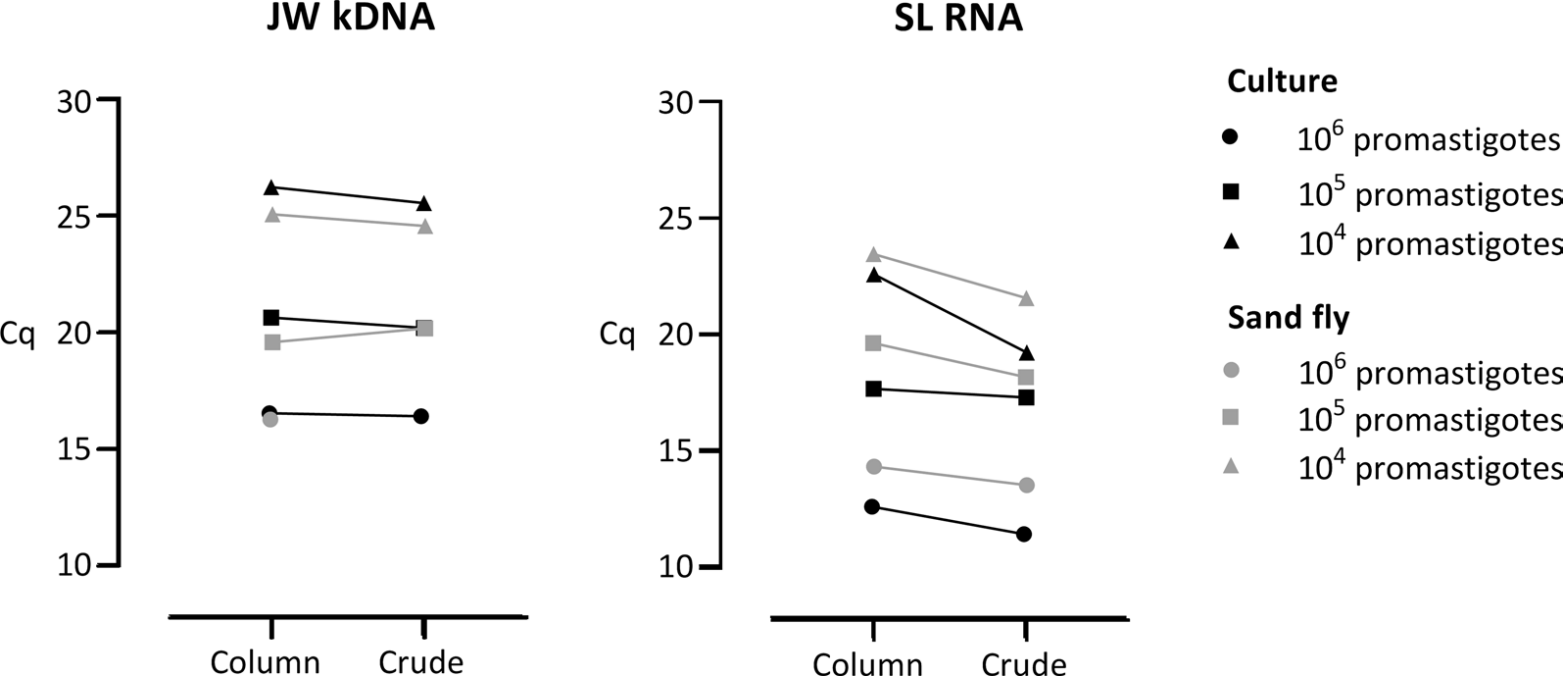
Extraction method and copy number comparison. Cq values of promastigotes isolated from culture (black symbols) and sand fly midguts (grey symbols) that were extracted with a commercial column extraction (‘column’) or crude high-salt extraction buffer (‘crude’) and subjected to JW kDNA and SL RNA qPCRs. Each symbol presents the assay result for a standardized concentration of promastigotes that was used for the comparisons. *Abbreviation*: Cq, quantification cycle

The crude extraction buffer with ethanol precipitation and column purification (respectively referred to as ‘crude’ and ‘column’ in Fig. 3) methods showed similar extraction efficiencies for kDNA, with comparable Cq values obtained for the standardized concentrations of promastigotes isolated from culture or sand fly midguts (Additional file 5: Data S4). Likewise, both methods performed well for SL RNA extraction, although the RNA yield appeared even slightly higher (on average 1.5 lower Cq values) with the crude method. The Cq values for kDNA were similar for promastigotes isolated from culture and sand fly midguts (Fig. 3, grey *versus* black symbols). For SL RNA, both extraction methods revealed that the Cq values for sand fly derived promastigotes were slightly but consistently higher (Cq on average 1.7) than those for culture-derived promastigotes. The JW kDNA qPCR reaction suffered inhibition in both runs for 10^6^ promastigotes isolated from sand fly midguts (Fig. 3, lacking grey circle for ‘crude’).

### Contribution of RNA *versus* DNA in the SL RNA qPCR assay

The differences in Cq values of the ten *L. major-*infected *L. longipalpis* nucleic acid extracts screened by the SL RNA qPCR with and without reverse transcriptase are presented in Table 1 (Additional file 6: Data S5). When the assay was performed without reverse transcriptase, the Cq values were on average 5.1 ± 1.1 higher than when the enzyme was used, meaning that only 3.8% (± 2.7%) of the fluorescence produced by the SL RNA qPCR assay was because of DNA amplification, while the remaining signal originated from SL RNA.

**Table 1:** Cq values of the SL RNA qPCR assay with and without reverse transcriptase enzyme

With reverse transcriptase (RNA + DNA)	Without reverse transcriptase (DNA)	Cq difference	% DNA
15.0	19.7	4.8	3.7
27.4	30.8	3.4	9.8
23.6	27.7	4.2	5.6
14.8	21.5	6.7	1.0
14.0	19.2	5.2	2.7
15.6	20.9	5.3	2.6
16.3	20.7	4.3	5.0
13.7	20.2	6.5	1.1
16.1	20.4	4.3	5.0
14.4	20.7	6.3	1.3
Mean ± SD		5.1 ± 1.1	3.8 ± 2.7

### LoD and LoQ of the SL RNA qPCR assay

**Fig. 4:**
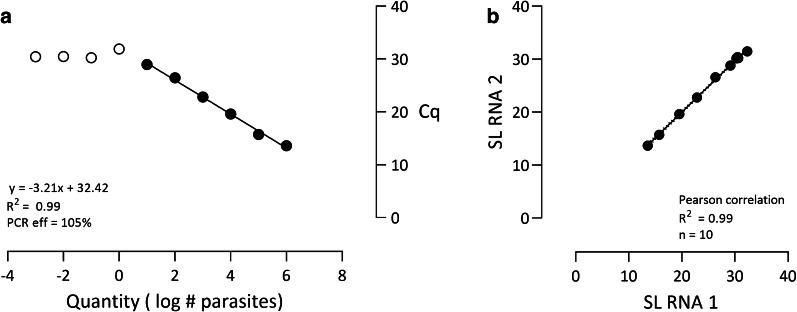
Performance of the SL RNA qPCR on a serial dilution of sand flies spiked with promastigotes. **a** Standard curve with linear regression and qPCR efficiency. The open symbols depict all concentrations that were detected by the assay, while the filled symbols are the parasite concentrations that show a linear correlation. **b** Inter-run variability of the SL RNA qPCR in two replicates, analyzed by Pearson’s correlation and linear regression analysis. Dotted lines indicate the 95% confidence intervals. *Abbreviation*: Cq, quantification cycle

Based on the serial dilution of *L. longipalpis* sand flies spiked with *L. major* promastigotes, the theoretical LoD of the SL RNA qPCR was 10^−3^ parasite equivalents (Fig. 4a, Additional file 7: Data S6). For 1.6 × 10^7^ promastigotes, the assay did not provide a result in any of the two independent runs, implying that there was PCR inhibition at this concentration. The assay showed a very good PCR efficiency of 105% for the serial dilution down to 10 parasites, representing the theoretical LoQ. The Pearson’s correlation demonstrated an excellent inter-run stability for the two independent runs of the SL RNA qPCR on the serial dilution (Fig. 4b; *R*^2^ = 0.99, *n* = 10).

## Discussion

For eco-epidemiological surveys, a large number of sand fly and potential reservoir samples need to be screened in order to find some *Leishmania-*positive specimens, because the infection prevalence is overall quite low, even in endemic areas [24, 26]. Therefore, researchers currently opt for sensitive, low-cost, high-throughput molecular screening methods for *Leishmania* detection in vectors and potential hosts. These molecular methods are often expensive TaqMan probe assays that target DNA sequences, which may persist for quite some time after parasite death [27]. In our study, we evaluated whether the recently developed SL RNA qPCR assay by Eberhardt et al. [23] enables *Leishmania* detection in sand flies and skin tissue from CL-infected animals. The targeted 39-bp SL RNA sequence is conserved amongst *Leishmania* species and fulfils an essential function in RNA trans-splicing and polyadenylation processes [28]. To our knowledge, this study is the first to evaluate the use of an RNA target for *Leishmania* detection in vectors and field-sampled tissue of reservoir hosts and to combine it with a low-cost extraction method. Since RNA quickly decays after death of the infectious agent, it is considered as a promising detection marker for viable *Leishmania* parasites [22, 29, 30], although the half-life of SL RNA as a small nuclear RNA molecule remains to be determined. We assessed the performance of the SL RNA qPCR assay in comparison with two other molecular diagnostic assays on field-collected and laboratory-infected sand flies that were extracted with the crude method and hyrax tissue samples. The JW kDNA [14, 24] and SL RNA qPCRs [23] showed concordant results using the laboratory *L. major*-infected *L. longipalpis* and field-collected *L. aethiopica*-infected *P. pedifer* sand flies. Both assays identified the same positive and negative samples, indicating that they have a similar analytical sensitivity and specificity. It was surprising that the SL RNA qPCR performed very well on field-collected sand flies, considering that these samples had not been preserved under favorable conditions for RNA, which may relate to the short amplicon length [31, 32]. These observations indicate that SL RNA could be an interesting target for *Leishmania* detection in vectors collected during entomological surveys.

On the contrary, the MP kDNA and *18S* DNA targets could not identify all positive laboratory-infected sand flies. One reason is the low copy number of the *18S* rDNA fragment (50–200 copies per *Leishmania* genome) [33] compared to the much higher copy number of SL RNA (a single *Trypanosoma* cell contains about 8600 copies [34]) and kDNA (a *Leishmania* parasite contains approximately 10,000 copies [35]). Whereas the Cq values for kDNA were fairly similar in promastigotes isolated from sand flies and from culture, Cq values for SL RNA were more distinct, suggesting that this marker is potentially slightly less abundant in parasites isolated from sand flies. This may indicate a reduced transcriptional activity of the vector-derived parasite pool (containing various life-cycle stages) as compared to culture-derived parasites.

**Fig. 5:**
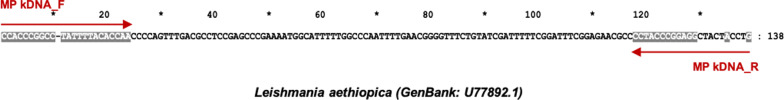
Annealing of MP kDNA qPCR primers to the *L. aethiopica* kDNA fragment (GenBank: U77892.1)

The duplex assay targeting the MP kDNA marker could not identify some of the laboratory-infected sand flies that were positive by the JW kDNA and SL RNA qPCRs and Cq values for the MP kDNA target were generally slightly higher than for the JW kDNA assay. This is most probably because multiplex qPCRs are commonly slightly less sensitive than uniplex assays and due to the intrinsic difference in fluorescent signal development between SYBR Green and TaqMan probe assays [36, 37]. Additionally, the qPCR targeting the MP kDNA marker resulted in higher Cq values on the field-collected sand flies (*L. aethiopica-*infected) compared to laboratory-infected sand flies (*L. tropica*), which most probably relates to mismatches of the reverse primer with the *L. aethiopica* kDNA fragment (Fig. 5) [17]. Earlier observations of a lower sensitivity for *L. tropica* (genetically very similar to *L. aethiopica*) and *L. mexicana* [23] corroborates the limitations of this MP kDNA target that was originally described by Mary et al. [17] for detection of *L. donovani*. The SL RNA qPCR provided equal Cq values for various *Leishmania* species, demonstrating its suitability as a pan-*Leishmania* assay [23].

Although only a few positive hyrax tissue samples were tested, the JW kDNA qPCR could identify most of the true-positive samples under the used sample storage conditions. A sample was considered positive if identified by two different assays. The SL RNA assay identified one false-negative sample and showed generally higher Cq values compared to the JW kDNA PCR than for sand fly screening, which is probably due to the fact that the samples had been stored in ethanol for two years before DNA/RNA extraction was performed. Most likely, proper RNA storage conditions and/or immediate RNA isolation would result in a substantially improved performance of the SL RNA qPCR on tissue samples [32, 34]. Favoring this viewpoint, the SL RNA qPCR showed excellent analytical sensitivity in laboratory-infected (*L. infantum*) mouse spleen and liver samples, detecting down to 10^−3^ parasite equivalents per mg tissue [23].

Considering the large sample size that needs to be screened in search for positive field specimens, a low-cost, efficient nucleic acid extraction method is preferred [9]. We found that a crude extraction buffer in combination with an ethanol precipitation step is as efficient as a commercial column extraction for downstream DNA and RNA detection in sand flies. Cq values tended to be even slightly lower when the extraction was carried out with the crude method, suggesting that there is some nucleic acid loss on the silica columns, or in addition, that some DNA is detected. Other important advantages of this crude extraction method are the low-cost and reduction in sample processing time as maceration is not required [8, 9]. The latter is compensated by a more time-consuming ethanol precipitation step. However, because of the low prevalence in field-collected sand flies, individual extracts can be pooled to reduce the number of samples for purification and PCR [24]. This method extracts all nucleic acids, including RNA and DNA. Nevertheless, we found that only a fraction of the generated signal of the SL RNA qPCR originates from DNA amplification, which is quite low considering the storage conditions and crude extraction method used. The actual contribution of RNA *versus* DNA in positive samples can be easily assessed by comparison with a no-reverse transcription control.

Determination of the parasite load in sand flies can be highly informative, especially for studies that investigate, e.g. the vectorial capacity. Previously, the LoD of the SL RNA qPCR on cultured promastigotes has been established at 0.0002 parasite equivalents [23]. We assessed the theoretical LoD of the SL RNA qPCR based on sand flies spiked with a serial dilution of *L. major* promastigotes. The determined theoretical LoD of 10^−3^ parasite equivalents per reaction of our assay is similar to findings of Bezerra-Vasconcelos et al. [13], who could detect 10^−3^ parasites per reaction with a kDNA qPCR assay on *L. infantum*-spiked *L. longipalpis* sand flies. This substantiates that the sensitivity of qPCR assays targeting SL RNA and kDNA are comparable, which corroborates the comparative assessment performed in the present study. Moreover, congruence of the assays appears very good, indicating that both can achieve reliable quantification. Based on the standard curve, it can be concluded that SL RNA qPCR can quantify down to 10 parasites per sand fly with high PCR efficiency, which is sufficient for determination of biologically relevant parasite loads.

## Conclusions

Overall, to the best of our knowledge, this study shows for the first time that the SL RNA target can be used for detection and quantification of *Leishmania* parasites in field-collected and laboratory-infected sand flies, even in combination with a crude, low-cost extraction method. The SL RNA qPCR assay is inexpensive, sensitive and pan-*Leishmania* specific, which can be a major advantage for eco-epidemiological studies including identification of vectors and reservoirs.

## Supplementary information

**Additional file 1: Table S1.** qPCR results of sand fly and hyrax samples from the laboratory and field in four different assays. For each assay the mean quantification cycle threshold value (± standard deviation) are presented.

**Additional file 2: Data S1.** Dataset assay comparison on laboratory-infected (*Leishmania major*) *Lutzomyia longipalpis* sand flies.

**Additional file 3: Data S2.** Dataset assay comparison on field-collected (Ethiopia) *Phlebotomus pedifer* sand flies (some *L. aethiopica*-infected).

**Additional file 4: Data S3.** Dataset assay comparison on field (Ethiopia) collected *Heterohyrax brucei* tissue samples (some *L. aethiopica-*infected).

**Additional file 5: Data S4.** Data of extraction method and copy number comparison.

**Additional file 6: Data S5.** Data of SL RNA qPCR with and without the use of reverse transcriptase.

**Additional file 7: Data S6.** Data of standard curve of the SL RNA qPCR based on sand flies spiked with promastigotes.

## Data Availability

All data generated and analyzed during this study are included within this published article and its additional files.

## Abbreviations

CL: cutaneous leishmaniasis
kDNA: kinetoplast DNA
qPCR: quantitative PCR
SL RNA: spliced leader RNA
Cq: quantification cycle
LoD: limit of detection
LoQ: limit of quantification
