# Supplementary material for: Evaluation of a pan-Leishmania SL RNA qPCR assay for parasite detection in laboratory-reared and field-collected sand flies and reservoir hosts
Source: Parasit Vectors. 2020 Jun 1;13:276. doi: 10.1186/s13071-020-04141-y (PMC7268266; doi:10.1186/s13071-020-04141-y)
Supplement: Supplementary file 1 — Additional file 1: Table S1. qPCR results of sand fly and hyrax samples from the laboratory and field in four different assays. For each assay the mean quantification cycle threshold value (± standard deviation) are presented. [file 13071_2020_4141_MOESM1_ESM.pdf]

	# positive tests	# samples	MP 18S qPCR	MP kDNA qPCR	JW kDNA qPCR	SL-RNA qPCR
A) Laboratory *Lutzomyia longipalpis* sand flies (infected with *Leishmania major*)						
	0	2				
	1	0				
	2	2			27.1 (± 0.2)	25.7 (± 0.7)
	3	10		21. 4 (± 4.4)	17.5 (± 3.1)	17.1 (± 2.4)
	4	82	30.3 (± 2.3)	17.3 (± 1.4)	14.6 (± 1.4)	13.8 (± 0.9)
B) Field *Phlebotomus pedifer* sand flies (infected with *L. aethiopica*)						
	0	20				
	1	0				
	2	0				
	3	0				
	4	17	24.4 (± 4.2)	22.9 (± 4.1)	13.7 (± 3.9)	14.7 (± 3.4)
C) Hyrax skin tissue (infected with *L. aethiopica*)						
	0	15	-			
	1	0	-			
	2	1	-	35.5	24.4	
		2	-		25.5 (± 0.8)	29.9 (± 3.3)
	3	4	-	32.6 (± 1.2)	16.6 (± 1.7)	27.5 (± 2.8)

+: positive; -: not tested with this assay; MP 18S qPCR: Multiplex TaqMan probe real-time PCR targeting 18S DNA; MP kDNA qPCR: Multiplex TaqMan probe real-time PCR targeting kDNA; JW kDNA qPCR: SYBR Green real-time PCR assay targeting kDNA; SL-RNA qPCR: SYBR Green real-time PCR targeting SL-RNA.
